# A functional endosomal pathway is necessary for lysosome biogenesis in *Drosophila*

**DOI:** 10.1186/s12860-016-0115-7

**Published:** 2016-11-16

**Authors:** Anne-Claire Jacomin, Marie-Odile Fauvarque, Emmanuel Taillebourg

**Affiliations:** 1 Université Grenoble-Alpes, F-38041 Grenoble, France; 2 CEA-DSV-iRTSV-BGE-Gen&Chem, 17, rue des Martyrs, 38054 Grenoble, Cedex 9 France; 3 INSERM, U1038, F-38054 Grenoble, France; 4 Present address: School of Life Sciences, University of Warwick, Coventry, UK

**Keywords:** Lysosomal biogenesis, Endosomal system, Endocytosis, Autophagy, Lysosome, *Drosophila melanogaster*

## Abstract

**Background:**

Lysosomes are the major catabolic compartment within eukaryotic cells, and their biogenesis requires the integration of the biosynthetic and endosomal pathways. Endocytosis and autophagy are the primary inputs of the lysosomal degradation pathway. Endocytosis is specifically needed for the degradation of membrane proteins whereas autophagy is responsible for the degradation of cytoplasmic components. We previously identified the deubiquitinating enzyme UBPY/USP8 as being necessary for lysosomal biogenesis and productive autophagy in *Drosophila.* Because UBPY/USP8 has been widely described for its function in the endosomal system, we hypothesized that disrupting the endosomal pathway itself may affect the biogenesis of the lysosomes.

**Results:**

In the present study, we blocked the progression of the endosomal pathway at different levels of maturation of the endosomes by expressing in fat body cells either dsRNAs or dominant negative mutants targeting components of the endosomal machinery: Shibire, Rab4, Rab5, Chmp1 and Rab7. We observed that inhibition of endosomal trafficking at different steps in vivo is systematically associated with defects in lysosome biogenesis, resulting in autophagy flux blockade.

**Conclusion:**

Our results show that the integrity of the endosomal system is required for lysosome biogenesis and productive autophagy in vivo.

**Electronic supplementary material:**

The online version of this article (doi:10.1186/s12860-016-0115-7) contains supplementary material, which is available to authorized users.

## Background

Lysosomes are the primary degradative organelles of the cell. They are found in virtually all eukaryotic cells and were initially described in the 1950s by the Nobel laureate Christian de Duve [1]. Their substrates include all kinds of macromolecules delivered either by endocytosis, phagocytosis or autophagy. Lysosomal biogenesis is orchestrated by the transcription factor EB (TFEB) which activates the transcription of ~500 target genes involved in lysosomal biogenesis and autophagy [2, 3]. On the other hand, lysosomal biogenesis also requires the integration of the endosomal and biosynthetic pathways: newly synthesized lysosomal proteins are delivered to lysosomes either directly from the *trans*-Golgi network to the endosomal system using the mannose-6-phosphate receptor (MPR) or the Vps41/VAMP7 pathway or indirectly via alternative receptors such as LIMP-2 [4–7]. In *Drosophila*, defects in the biogenesis of lysosomes and lysosomes related organelles such as eye pigment granules result in defective eye pigmentation which has led to the identification of the “granule group” proteins including Deep-orange, homologue of Vps18p, Carnation, homologue of Vps33A and Light, homologue of Vps41 [8–11].

The endosomal system constitutes a network of progressively maturing vesicles that is required, among other physiological functions, for the degradation of membrane proteins such as receptors and ionic channels. These proteins enter the endosomal system through clathrin or caveolin-coated vesicles and are then delivered to early endosomes. From here, membrane proteins can either be recycled to the plasma membrane or directed for degradation via the multivesicular bodies (MVB) to late endosomes that eventually fuse with lysosomes [12]. Sorting to the MVB requires the ESCRT (Endosomal Sorting Complex Required for Transport) machinery composed of four distinct complexes called ESCRT-0 to –III. Apart from ESCRT machinery, progression along the endosomal pathway requires the activity of Rab GTPases: Rab5 is located to the clathrin coated vesicles and early endosomes and contributes to endocytic internalization and early endosome fusion [13, 14]; Rab4 is located at the early and recycling endosomes, and is involved in the recycling to plasma membrane [15]; Rab7 is involved in the transport from early to late endosomes and is an essential component of the lysosomes biogenesis and maintenance [5, 16, 17]. Rab GTPases notably recruit tethering and docking machinery to bring membranes closer, after which the SNARE proteins complete the fusion process [12].

We have previously observed that the deubiquitinating enzyme UBPY is required for lysosomal biogenesis in *Drosophila* [18]. However, UBPY is mainly known for playing an important role in the sorting of many membrane receptors in *Drosophila* [19, 20] and mammalian cells [21–26]. Given the integration of lysosomal biogenesis and the endosomal system, we hypothesize that the lysosomal defects observed in UBPY mutant cells might be a consequence of UBPY function in the endosomal system and seek to further test the requirement of ongoing endosomal trafficking for lysosomal biogenesis in vivo. In the present report, we show that inhibition of endosomal trafficking at different steps is associated with defects in lysosomal biogenesis and blockade of autophagic degradation indicating that a functional endosomal system is required for lysosome biogenesis in vivo.

## Results

### Endosomal trafficking is required for lysosomal biogenesis

In order to evaluate the effect of the disruption of the endosomal trafficking on the formation of the lysosome, we affected the function of key players of the endosomal system by expressing dsRNAs or dominant-negative mutants targeting them. To circumvent any potential detrimental effects at the tissue or organism levels, the FLPout method [27] was used to express transgenes in a few fat body cells surrounded by wild-type cells (see Methods and Additional file 1: Figure S1). The transgenes used were: a dominant negative form of Shibire – the *Drosophila* homologue of the Dynamin GTPase that is required for the scission of the newly formed endosomes from the plasma membrane – (ShiK44A) which blocks the budding of endocytic vesicles from the plasma membrane [28], a dsRNA targeting *Rab5* [14] that efficiently inhibits the early endosomal Rab5 protein (Additional file 2: Figure S2), a dominant negative mutant of Rab4 (Rab4SN) which blocks the endosomal recycling pathway [15] and a dsRNA against *Chmp1* – a component of the ESCRT machinery – which impairs the formation of intraluminal vesicles in the MVB [29–31]. Lastly, a dominant negative mutant of Rab7 (Rab7TN) was added as a control because Rab7 is essential for lysosomes biogenesis and maintenance of the perinuclear lysosome compartment [17, 32, 33]. The ability of these transgenes to efficiently affect the endosomal process was assessed by monitoring the endocytic uptake of the fluid phase marker Texas Red-avidin (Additional file 3: Figure S3).

**Fig. 1:**
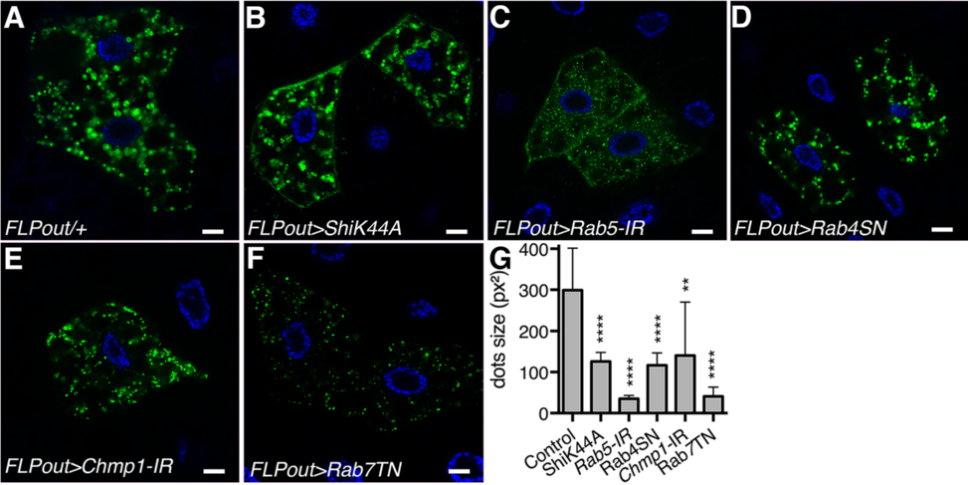
Defects in the endosomal pathway affect the size of LAMP1-positive lysosomes. **a-f** Confocal sections of larval fat bodies clonally expressing the lysosomal marker GFP-LAMP1 (*green*) alone (**a**) or in combination with the dominant negative or silencing transgenes for *Shibire* (**b**), *Rab5* (**c**), *Rab4* (**d**), *Chmp1* (**e**) or *Rab7* (**f**). Fixed fat bodies were stained with Hoechst (*blue*). Scale bar: 10 μm. **g** Quantification of GFP-LAMP1 dots size. *Bars* denote mean ± s.d. Statistical significance was determined using one-way ANOVA: *p < 0.05, **p < 0.005, ***p < 0.0005, ****p < 0.0001. Genotypes: **a** *y w hs-FLP/+; UAS-GFP-LAMP1/+; Ac > CD2 > Gal4/+*, **b** *y w hs-FLP/UAS-ShiK44A; UAS-GFP- LAMP1/+; Ac > CD2 > Gal4/ UAS-ShiK44A*, **c** *y w hs-FLP/+; UAS-GFP-LAMP1/+; Ac > CD2 > Gal4/UAS-Rab5-IR*, **d** *y w hs-FLP/+; UAS-GFP-LAMP1/+; Ac > CD2 > Gal4/UAS-Rab4SN*, **e** *y w hs-FLP/+; UAS-GFP-LAMP1/+; Ac > CD2 > Gal4/UAS-Chmp1-IR*, **f** *y w hs-FLP/+; UAS-GFP-LAMP1/UAS-Rab7TN; Ac > CD2 > Gal4/+*

To visualize the lysosomes, we first used one of the most abundant lysosomal membrane protein as a marker: the lysosomal-associated membrane protein 1 (LAMP1). The GFP-LAMP1 transgene used in these experiments consists in the fusion between eGFP and the transmembrane domain and cytoplasmic tail derived from human LAMP1 [34]. In wild-type cells, the GFP-LAMP1 fusion protein identified large perinuclear vesicles corresponding to lysosomes as well as smaller vesicles evenly distributed in the cytoplasm (Fig. 1a). As expected, in cells expressing the Rab7 dominant-negative protein (Rab7TN), the large perinuclear lysosomes were missing whereas smaller dots were still present (Fig. 1f). This observation is in agreement with previous reports showing that Rab7 is essential for lysosomes biogenesis and maintenance of the perinuclear lysosome compartment [17, 32, 33]. Interestingly, whenever endosomal trafficking has been affected using either dsRNAs targeting *Rab5* and *Chmp1* or dominant negative mutants interfering with Shibire and Rab4 the size of the GFP-LAMP1 vesicles was significantly reduced (Fig. 1b-e, g). These results thus show that inhibition of endosomal trafficking results in a reduction of the size of lysosomes.

**Fig. 2:**
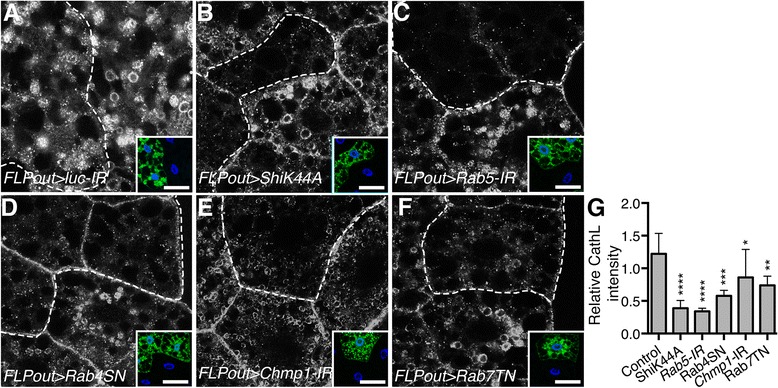
Defects in the endosomal pathway affect the distribution of the lysosomal hydrolase Cathepsin L. **a-f** Confocal sections of larval fat bodies with control clonal cells (**a**) or clonally expressing the dominant negative or silencing transgenes for *Shibire* (**b**), *Rab5* (**c**), *Rab4* (**d**), *Chmp1* (**e**) or *Rab7* (**f**). Fixed fat bodies were stained for the endogenous lysosomal hydrolase Cathepsin L. Clonal cells are outlined with a *dotted line* using the GFP-LAMP1 reporter also expressed by these cells as shown in the inset. Scale bar: 10 μm. **g** Quantification of the mean relative intensity of the Cathepsin L staining in transgene expressing cells compared to the staining intensity of the adjacent wild-type neighboring cells. Bars denote mean ± s.d. Statistical significance was determined using one-way ANOVA: *p < 0.05, **p < 0.005, ***p < 0.0005, ****p < 0.0001. Genotypes: **a** *y w hs-FLP/UAS-ShiK44A; UAS-GFP-LAMP1/+; Ac > CD2 > Gal4/UAS-lucIR*, **b** *y w hs-FLP/UAS-ShiK44A; UAS-GFP- LAMP1/+; Ac > CD2 > Gal4/ UAS-ShiK44A*, **c** *y w hs-FLP/+; UAS-GFP-LAMP1/+; Ac > CD2 > Gal4/UAS-Rab5-IR*, **d** *y w hs-FLP/+; UAS-GFP-LAMP1/+; Ac > CD2 > Gal4/UAS-Rab4SN*, **e** *y w hs-FLP/+; UAS-GFP-LAMP1/+; Ac > CD2 > Gal4/UAS-Chmp1-IR*, **f** *y w hs-FLP/+; UAS-GFP-LAMP1/UAS-Rab7TN; Ac > CD2 > Gal4/+*

Lysosomes contain many acid hydrolases, including cathepsins that are responsible for their catabolic ability. Most of these enzymes are synthesized in the endoplasmic reticulum, sorted in the Golgi apparatus using the mannose-6-phosphate receptor (MPR) and delivered to late endosomes [5]. Yet, MPR-independent routes have also been described for their delivery to the lysosomes [4, 7]. In order to evaluate the lysosomal activity, we have compared the distribution of the lysosomal hydrolase Cathepsin L between control cells and cells where the endosomal flux is disrupted. In wild-type cells, sizeable lysosomes were readily identified and the overall Cathepsin L staining intensity was similar between cells expressing an RNAi transgene targeting Luciferase as no-target control and wild-type neighboring cells (Fig. 2a, g). On the other hand, as expected, cells expressing the Rab7 dominant-negative protein showed a drastic change in Cathepsin L distribution: the lysosomes were missing, and the overall Cathepsin L staining intensity was decreased (Fig. 2f, g). Strikingly, the same observations were made in cells where endosomal trafficking is inhibited using either dsRNAs targeting *Rab5* and *Chmp1* or dominant negative mutants interfering with Shibire and Rab4 (Fig. 2b-e, g). These results, combined with the previous data obtained with the GFP-LAMP1 protein, thus indicate that inhibition of endosomal trafficking in vivo affects lysosomal biogenesis.

### Endosomal trafficking is required for productive autophagy

Autophagy is another conserved catabolic process allowing for the degradation of cytoplasmic constituents by the lysosomes. To be degraded, autophagosomes have to deliver their contents to lysosomes. Lysosomes are thus key players in this process, and any disturbance of their function or biogenesis potentially affects autophagy [35].

**Fig. 3:**
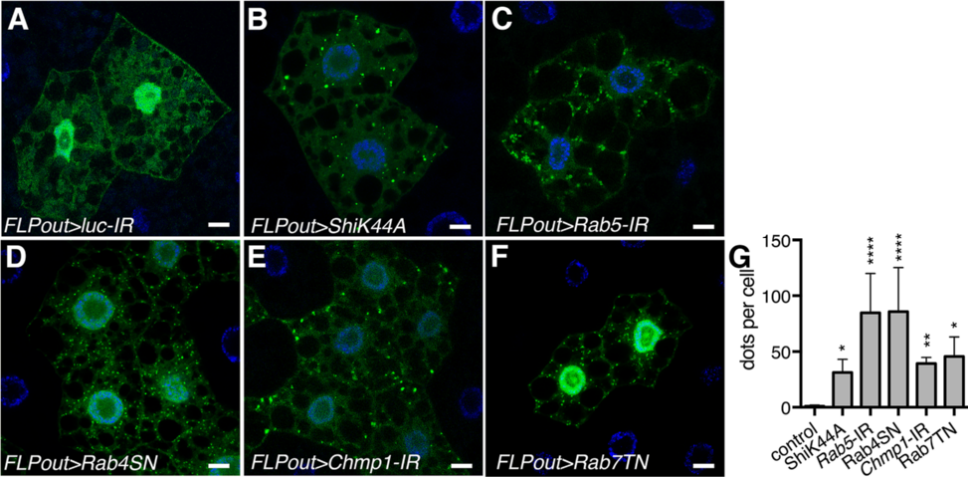
Blocking the endosomal pathway induces the accumulation of autophagosomes. **a-f** Confocal sections of larval fat bodies clonally expressing the autophagy marker GFP-Atg8a (*green*) alone (**a**) or in combination with the dominant negative or silencing transgenes for *Shibire* (**b**), *Rab5* (**c**), *Rab4* (**d**), *Chmp1* (**e**) or *Rab7* (**f**). Fixed fat bodies were stained with Hoechst (*blue*). Scale bar: 10 μm. **g** Quantification of the number of GFP-Atg8a dots per cells. *Bars* denote mean ± s.d. Statistical significance was determined using one-way ANOVA: *p < 0.05, **p < 0.005, ***p < 0.0005, ****p < 0.0001. Genotypes: **a** *y w hs-FLP/+; UAS-GFP-Atg8a/+; Ac > CD2 > Gal4/+*, **b** *y w hs-FLP/UAS-ShiK44A; UAS-GFP- Atg8a/UAS-LucIR; Ac > CD2 > Gal4/ UAS-ShiK44A*, **c** *y w hs-FLP/+; UAS-GFP- Atg8a/+; Ac > CD2 > Gal4/UAS-Rab5-IR*, **d** *y w hs-FLP/+; UAS-GFP- Atg8a/+; Ac > CD2 > Gal4/UAS-Rab4SN*, **e** *y w hs-FLP/+; UAS-GFP- Atg8a/+; Ac > CD2 > Gal4/UAS-Chmp1-IR*, **f** *y w hs-FLP/+; UAS-GFP- Atg8a/UAS-Rab7TN; Ac > CD2 > Gal4/+*

To investigate autophagy, we first used the GFP-tagged Atg8a marker. In fed condition, Atg8a is distributed throughout the cytoplasm and accumulates in the nucleus (Fig. 3a), whereas upon autophagy induction by starvation, Atg8a is exported from the nucleus and resides in the cytoplasm where it is recruited to the autophagosomes [36] (data not shown). We observed that, even in fed condition, cells expressing transgenes resulting in inhibition of endosomal trafficking displayed numerous GFP-Atg8a positive autophagosomes (Fig. 3b-g).

**Fig. 4:**
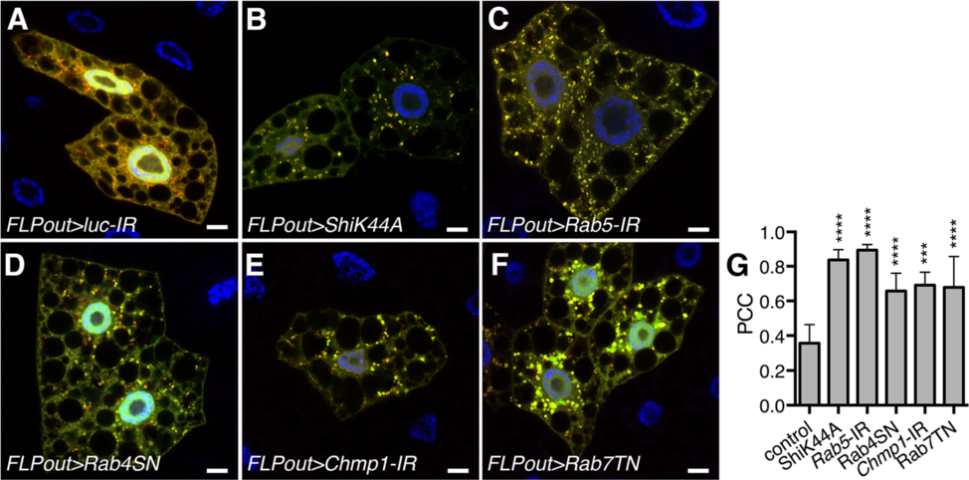
Defects in the endosomal pathway result in a blockade of the autophagy flux. **a-f** Confocal sections of larval fat bodies clonally expressing the autophagy flux marker GFP-mCherry-Atg8a (*green*) alone (**a**) or in combination with the dominant negative or silencing transgenes for *Shibire* (**b**), *Rab5* (**c**), *Rab4* (**d**), *Chmp1* (**e**) or *Rab7* (**f**). Fixed fat bodies were stained with Hoechst (*blue*). Scale bar: 10 μm. **g** Quantification of the colocalization of mCherry and GFP signals using the Pearson’s correlation coefficient (PCC). *Bars* denote mean ± s.d. Statistical significance was determined using one-way ANOVA: *p < 0.05, **p < 0.005, ***p < 0.0005, ****p < 0.0001. Genotypes: **a** *y w hs-FLP/+; UAS-GFP-mCherry-Atg8a/+; Ac > CD2 > Gal4/UAS-lucIR*, **b** *y w hs-FLP/UAS-ShiK44A; UAS-GFP-mCherry-Atg8a/+; Ac > CD2 > Gal4/ UAS-ShiK44A*, **c** *y w hs-FLP/+; UAS-GFP-mCherry-Atg8a/+; Ac > CD2 > Gal4/UAS-Rab5-IR*, **d** *y w hs-FLP/+; UAS-GFP-mCherry-Atg8a/+; Ac > CD2 > Gal4/UAS-Rab4SN*, **e** *y w hs-FLP/+; UAS-GFP-mCherry-Atg8a/+; Ac > CD2 > Gal4/UAS-Chmp1-IR*, **f** *y w hs-FLP/+; UAS-GFP-mCherry-Atg8a/UAS-Rab7TN; Ac > CD2 > Gal4/+*

Accumulation of autophagosomes can either be a consequence of *de novo* autophagosomes formation due to autophagy induction or of autophagosomal degradation defects inducing a blockade of the autophagy flux. To distinguish between these possibilities, we first made use of a transgene expressing the GFP-mCherry-Atg8a fusion protein [37]. The merged signal between GFP and mCherry (yellow) fluorescence is representative of autophagosomes while only red fluorescence is characteristic of autolysosomes due to quenching of the GFP fluorescence in these acidic structures. As expected, wild-type fat body cells in which autophagy was activated by starving the larvae for 4 h in a solution of 20% sucrose, showed yellow and red vesicles (Fig. 4a) corresponding to autophagosomes and autolysosomes, respectively. In contrast, in fat bodies from fed larvae, cells expressing transgenes affecting endosomal trafficking displayed mainly yellow vesicles (Fig. 4b-g) indicating the presence of autophagosomes but a lack of autolysosomes in basal condition. These results indicate that the accumulation of autophagosome in these cells results from a blockade of the autophagy flux. To further investigate this, we used the membrane-permeable vital dye Lysotracker-Red that stains acidic vesicles and whose staining intensity is drastically increased in wild-type starved larvae due to the accumulation of large autolysosomes. The staining of fat bodies from starved larvae demonstrated a reduction in the intensity of the Lysotracker-Red staining in the cells expressing the transgenes affecting endosomal trafficking compared to the wild-type neighboring cells (Additional file 4: Figure S4). Altogether, these observations suggest that not only basal autophagy flux (fed condition) but also starvation-induced autophagy is impaired in these cells.

**Fig. 5:**
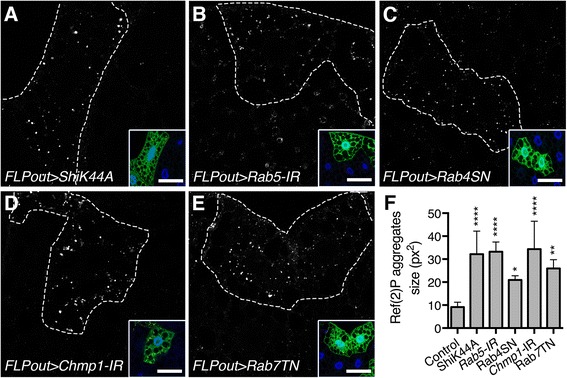
Defects in the endosomal pathway affect the degradation of the autophagy substrate Ref(2)P/p62. **a-e** Confocal sections of larval fat bodies clonally expressing the dominant negative or silencing transgenes for *Shibire* (**a**), *Rab5* (**b**), *Rab4* (**c**), *Chmp1* (**d**) or *Rab7* (**e**). Fixed fat bodies were stained for the endogenous Ref(2)P/p62 protein. Clonal cells are outlined with a *dotted line* using the GFP-Atg8a reporter also expressed by these cells as shown in the inset. Scale bar: 10 μm. **f** Quantification of the size of the Ref(2)P/p62 aggregates in transgene expressing cells compared to the adjacent wild-type neighboring cells. *Bars* denote mean ± s.d. Statistical significance was determined using one-way ANOVA: *p < 0.05, **p < 0.005, ***p < 0.0005, ****p < 0.0001. Genotypes: **a** *y w hs-FLP/UAS-ShiK44A; UAS-GFP-Atg8a/+; Ac > CD2 > Gal4/ UAS-ShiK44A*, **b** *y w hs-FLP/+; UAS-GFP- Atg8a/+; Ac > CD2 > Gal4/UAS-Rab5-IR*, **c** *y w hs-FLP/+; UAS-GFP- Atg8a/+; Ac > CD2 > Gal4/UAS-Rab4SN*, **d** *y w hs-FLP/+; UAS-GFP- Atg8a/+; Ac > CD2 > Gal4/UAS-Chmp1-IR*, **e** *y w hs-FLP/+; UAS-GFP- Atg8a/UAS-Rab7TN; Ac > CD2 > Gal4/+*

Monitoring the autophagy flux can also be done by assessing the degradation of known autophagic substrates which accumulate when the autophagy flux is blocked [38, 39]. We observed the accumulation of Ref(2)P, the *Drosophila* homolog of the autophagy receptor p62 [38, 40], in cells expressing the transgenes affecting endosomal trafficking compared to wild-type neighboring cells (Fig. 5). Altogether these results thus show that inhibition of endosomal trafficking results in autophagy flux blockade indicating that the lysosomal function is affected.

## Discussion

We previously identified UBPY as a new deubiquitinating enzyme affecting lysosomal biogenesis in *Drosophila* [18]. Earlier studies extensively showed the implication of UBPY in the endosomal pathway in both *Drosophila* and mammalian cultured cell models [19–26, 41, 42]. We hypothesized that the autophagy flux blockade and impaired lysosomes formation induced by *Ubpy* loss-of-function might be related to its function in the endosomal pathway, suggesting that the overall endosomal process is crucial for lysosomal biogenesis. In the present report, we have investigated this hypothesis by inhibiting endosomal trafficking at different steps – from the plasma membrane to the endo-lysosomal compartment. Using lysosomal markers such as the lysosomal membrane protein LAMP1 and the lysosomal hydrolase Cathepsin L, we observed that inhibition of endosomal trafficking consistently resulted in severe lysosomal biogenesis defects. Besides, the autophagic process in the cells presenting a defective endosomal trafficking was constitutively impaired, as revealed by the use of the GFP- and tandem GFP-mCherry-tagged Atg8a transgenes, and the accumulation of the autophagy substrate Ref(2)P/p62. Altogether, our results show that a functional endosomal pathway is required for lysosomal biogenesis and, as a consequence, for productive autophagy.

To date, two alternative models for lysosome biogenesis have been proposed [43]. In the maturation model, endosomes are gradually transformed into lysosomes by the addition (delivery of lysosomal enzymes and membrane proteins from the Golgi apparatus) and removal (by recycling vesicles) of molecules. According to this model, lysosomes would not form without endosomal trafficking. A second model, the vesicular transport model, postulates that endosomes, late endosomes, and lysosomes are stable pre-existing compartments that communicate by continuous rounds of fusion and fission. Although studies in cultured cells are numerous and sometimes contradictory, in vivo evidence supporting any of these models are surprisingly scarce. To our knowledge, Rab5 is the only known endocytic protein whose inactivation has been shown to impair the biogenesis of the endo-lysosomal system in vivo [44]. Our results thus confirm the crucial role of Rab5 but also extend this property to other components of the endosomal process, actively supporting the maturation model: fully functional lysosomes are not pre-existing compartments, but instead result from the gradual maturation of endosomes to which lysosomal enzymes are delivered.

Furthermore, it has been shown that the endosomal and autophagy pathways share several components [45, 46]. In particular, the endosomal Rab5 protein has also been proposed to act at an early stage of autophagy since inhibition of Rab5 activity by overexpression of a dominant negative mutant decreases the number of autophagosomes in cultured mammalian cells [47]. This observation does not fit with ours indicating that autophagosomes accumulate in fat body cells silenced for *Rab5*. It is possible that the role of Rab5 in autophagy may be unique to mammals and not conserved in *Drosophila*. Alternatively, differences in the experimental systems (transient overexpression of a dominant negative form of Rab5 in cultured cells *versus* clonal impairment in a wild-type organ during larval development) may be at stake. A careful comparison of the autophagic phenotype induced by Rab5 inhibition or silencing in the same experimental model should resolve this point. It is worth noting that the scientific literature is quite contradictory on the requirement of endosomal pathway members for autophagy. Autophagosomes and ubiquitinated protein aggregates have been observed in ESCRT mutant cells [46, 48], indicating a blockade of autophagic degradation after autophagosomes formation in agreement with our results. In contrast, other studies have shown that perturbations of the endosomal pathway impair autophagosome formation in cultured cells [49–51].

## Conclusion

Our results demonstrated that genetic impairment of endosomal trafficking induces lysosomal defects in the widely used *Drosophila* fat body model. We further show that endosomal trafficking – because of its requirement for lysosomal biogenesis – is also required for efficient autophagic degradation. Indeed, these last years, the connection between lysosome biogenesis or function and autophagy has been extensively described, and an increasing body of evidence implicates defective autophagy in the ethology of lysosomal storage disorders, a group of approximately 50 rare inherited metabolic disorders that result from defects in lysosomal function. For example, stalled or blocked autophagy has been observed in the lipid storage disorder Niemann-Pick type C1 (NPC1) disease [52] and in the Gaucher disease, the most prevalent lysosomal storage disorder [53]. Moreover, regulation of these two processes is coordinated by the transcription factor EB (TFEB) which drives expression of autophagy and lysosomal genes [3]. By suggesting that these disorders can originate from defects in the endosomal system, our results thus open new avenues in the understanding of lysosomal storage diseases and of the numerous pathologies linked to autophagy deficiencies.

## Methods

### Drosophila stocks and clonal analysis

Flies were reared at 25 °C on standard cornmeal–yeast medium. The *UAS-Rab4SN*, *UAS-Rab5SN* and *UAS-Rab7TN* flies were provided by Dr Emery [54]. The *UAS-ShiK44A* (#5811), *UAS-Chmp1-IR* (#28906), UAS-*GFP-LAMP1* (#42714) [34] and *UAS-GFP-mCherry-Atg8a* (#37749) strains were obtained from the Bloomington *Drosophila* Stock Center and the *UAS-Rab5-IR* (#103945) strain from the Vienna *Drosophila* Resource Center. The *UAS-GFP-Atg8a* strain has been provided by Dr T. Neufeld. The *UAS-lucIR* line (#31603) was obtained from the Bloomington Drosophila Stock Center and corresponds to an RNAi targeting the *Luciferase* gene used as no target control.

For the FLPout GAL4/UAS method (Additional file 1: Figure S1), a FRT-flanked cassette blocking expression of the GAL4 gene is excised upon heat-shock induced expression of the FLP recombinase. This mitotic recombination event leads to the expression of the GAL4 gene and is transmitted across mitosis, generating clones of cells in which GAL4 expression is activated. These cells are identified by the expression of the fluorescent tagged-transgenes GFP-LAMP1, GFP-Atg8a or GFP-mCherry-Atg8a. Spontaneous activation of the Gal4 transcription factor has been reported and allows for the induction of Gal4 expressing cells without heat shock [55].

### Immunocytochemistry and microscopy

For the starvation experiments, young third instar larvae were washed twice in deionized water and placed for 4 h either on a regular diet medium (fed condition) or in a filtered solution of 20% sucrose in PBS to induce autophagy (starved condition) [56]. Antibody and phalloidin staining were performed as described previously [57]. The samples were imaged with a 63x magnification (oil immersion) using a Leica TCS-SP2 confocal microscope and the LCS software. The primary antibodies used in this study were the following: rabbit polyclonal against *D. melanogaster* Ref(2)P protein [58], and rabbit monoclonal anti-Cathepsin L (ab133641, Abcam). The appropriate Cy3-conjugated secondary antibodies were purchased from Jackson Immunoresearch Laboratories.

Lysotracker-Red staining on fat bodies was performed as in ref. [56]. Images were obtained with a fluorescence microscope (Nikon Eclipse 90i) controlled by Nikon Software (Universal Imaging Corp.) using a 60x Plan-Neofluor oil objective.

### Image analysis and processing

Image analysis was done with the Fiji/ImageJ software (National Institute of Health) [59]. The number of GFP-Atg8a dots and the size of the GFP-LAMP1 dots were determined using a semi-automated macro that allows for the identification, numbering and measuring of the dots whilst excluding the potential nuclear staining [60]. The quantitative analysis of the colocalization between the green and red dots using the GFP-mCherry-Atg8a construct was done with the JACoP plugin and represented by the Pearson’s correlation coefficient (PCC) [61]. Image processing was done with Photoshop CC 2014 (Adobe). All the pictures shown are representative of the whole tissue and of the observations made from different animals.

### Statistical analysis

Statistical analyses were performed using Prism 6 (GraphPad). *one-way ANOVA* with the *Dunnett’s test* for multiple comparisons has been used for the comparison of three or more groups.

## Additional files

Additional file 1: Figure S1. The FLPout system in *Drosophila*. (A-B) The recombination between FRT sites by the FLP recombinase under the control of a heat shock promoter (A) results in excision of the CD2- STOP cassette and expression of GAL4 (B) which in turn activates the expression of the transgenes downstream the UAS promoter, including a fluorescent reporter (GFP, or GFP-tagged protein). No recombination between the FRT leaves the CD2-STOP cassette in place, thus preventing GAL4 expression (C). (TIF 676 kb)
Additional file 2: Figure S2. Validation of the *Rab5-IR* transgene by immunofluorescence. Confocal sections of larval fat bodies clonally expressing the RNAi against *Rab5* stained for Rab5 (red). Fixed fat bodies were additionally stained with Hoechst (blue). The silenced cells were identified by the expression of the GFP-Atg8a transgene (green). Genotype: *y w hs-FLP/+; UAS-GFP- Atg8a/+; Ac > CD2 > Gal4/UAS-Rab5-IR. (TIF 1509 kb)*

Additional file 3: Figure S3. Validation of defects in the endosomal pathway by Texas Red-Avidin uptake. (A-B) The endocytic tracer TR-avidin fails to be internalized in clonal cells expressing either ShiK44A (A) or *Rab5-IR* (B). Clones were detected by the co-expression of the autophagy marker GFP-Atg8a. (C-G) Internalized TR-avidin fails to be transported to the lysosomes when late stages of the endocytic process are defective. Clonal cells were detected by the expression of the lysosomal marker GFP-LAMP1. Occasional colocalization between the endocytic tracer TR-avidin and the lysosomes are observed in control cells (D) but not in cells expressing Rab4SN (E), *Chmp1-IR* (F) or Rab7TN (G). Quantification of the colocalization between the TR-avidin and GFP-LAMP1 using the Pearson’s Correlation Coefficient (PCC) is shown in C. Bars denote mean ± s.d. Statistical significance was determined using one-way ANOVA: **p* < 0.05, ***p* < 0.005, ****p* < 0.0005, *****p* < 0.0001. Genotypes: (A) *y w hs-FLP/UAS-ShiK44A; UAS-GFP-Atg8a/+; Ac > CD2 > Gal4/ UAS-ShiK44A*, (B) *y w hs-FLP/+; UAS-GFP-Atg8a/+; Ac > CD2 > Gal4/UAS-Rab5-IR*, (C) *y w hs-FLP/+; UAS-GFP-LAMP1/+; Ac > CD2 > Gal4/+*, (D) *y w hs-FLP/+; UAS-GFP-LAMP1/+; Ac > CD2 > Gal4/UAS-Rab4SN*, (E) *y w hs-FLP/+; UAS-GFP-LAMP1/+; Ac > CD2 > Gal4/UAS-Chmp1-IR*, (F) *y w hs-FLP/+; UAS-GFP-LAMP1/UAS-Rab7TN; Ac > CD2 > Gal4/+. (TIF 1831 kb)*

Additional file 4: Figure S4. The endosomal pathway is required for the starvation-induced acidification of the lysosomes. Larvae clonally expressing the dominant negative or silencing transgenes for *Shibire* (A), *Rab5* (B), *Rab4* (C), *Chmp1* (D) or *Rab7* (E) were starved to induce autophagy and the acidification of the lysosomes. Alive fat bodies were stained with Lysotracker-Red. Clonal cells were identified by the expression of the GFP-Atg8a transgene. Genotypes: (A) *y w hs-FLP/UAS-ShiK44A; UAS-GFP-Atg8a/+; Ac > CD2 > Gal4/ UAS-ShiK44A*, (B) *y w hs-FLP/+; UAS-GFP- Atg8a/+; Ac > CD2 > Gal4/UAS-Rab5-IR*, (C) *y w hs-FLP/+; UAS-GFP- Atg8a/+; Ac > CD2 > Gal4/UAS-Rab4SN*, (D) *y w hs-FLP/+; UAS-GFP- Atg8a/+; Ac > CD2 > Gal4/UAS-Chmp1-IR*, (E) *y w hs-FLP/+; UAS-GFP- Atg8a/UAS-Rab7TN; Ac > CD2 > Gal4/+. (TIF 791 kb)*

## Abbreviations

Chmp1: Charged multivesicular body protein 1
dsRNA: Double-stranded RNA
ESCRT: Endosomal Sorting Complex Required for Transport
IR: Inverted repeat
LAMP1: Lysosomal-associated membrane protein 1
MVB: Multivesicular bodies
Ref(2)P: Refractory to Sigma P

## References

[1] de Duve C, Hayashi T (1959). Lysosomes, a new group of cytoplasmic particles. Subcellular particles.

[2] Palmieri M, Impey S, Kang H, di Ronza A, Pelz C, Sardiello M, Ballabio A (2011). Characterization of the CLEAR network reveals an integrated control of cellular clearance pathways. Hum Mol Genet.

[3] Settembre C, Di Malta C, Polito VA, Garcia Arencibia M, Vetrini F, Erdin S, Erdin SU, Huynh T, Medina D, Colella P (2011). TFEB links autophagy to lysosomal biogenesis. Science.

[4] Coutinho MF, Prata MJ, Alves S (2012). A shortcut to the lysosome: the mannose-6-phosphate-independent pathway. Mol Genet Metab.

[5] Saftig P, Klumperman J (2009). Lysosome biogenesis and lysosomal membrane proteins: trafficking meets function. Nat Rev Mol Cell Biol.

[6] Reczek D, Schwake M, Schroder J, Hughes H, Blanz J, Jin X, Brondyk W, Van Patten S, Edmunds T, Saftig P (2007). LIMP-2 is a receptor for lysosomal mannose-6-phosphate-independent targeting of beta-glucocerebrosidase. Cell.

[7] Pols MS, van Meel E, Oorschot V, ten Brink C, Fukuda M, Swetha MG, Mayor S, Klumperman J (2013). hVps41 and VAMP7 function in direct TGN to late endosome transport of lysosomal membrane proteins. Nat Commun.

[8] Sriram V, Krishnan KS, Mayor S (2003). deep-orange and carnation define distinct stages in late endosomal biogenesis in Drosophila melanogaster. J Cell Biol.

[9] Akbar MA, Ray S, Kramer H (2009). The SM protein Car/Vps33A regulates SNARE-mediated trafficking to lysosomes and lysosome-related organelles. Mol Biol Cell.

[10] Sevrioukov EA, He JP, Moghrabi N, Sunio A, Kramer H (1999). A role for the deep orange and carnation eye color genes in lysosomal delivery in Drosophila. Mol Cell.

[11] Warner TS, Sinclair DA, Fitzpatrick KA, Singh M, Devlin RH, Honda BM (1998). The light gene of Drosophila melanogaster encodes a homologue of VPS41, a yeast gene involved in cellular-protein trafficking. Genome.

[12] Schmidt MR, Haucke V (2007). Recycling endosomes in neuronal membrane traffic. Biol Cell.

[13] Bucci C, Parton RG, Mather IH, Stunnenberg H, Simons K, Hoflack B, Zerial M (1992). The small GTPase rab5 functions as a regulatory factor in the early endocytic pathway. Cell.

[14] Morrison HA, Dionne H, Rusten TE, Brech A, Fisher WW, Pfeiffer BD, Celniker SE, Stenmark H, Bilder D (2008). Regulation of early endosomal entry by the Drosophila tumor suppressors Rabenosyn and Vps45. Mol Biol Cell.

[15] Sönnichsen B, De Renzis S, Nielsen E, Rietdorf J, Zerial M (2000). Distinct membrane domains on endosomes in the recycling pathway visualized by multicolor imaging of Rab4, Rab5, and Rab11. J Cell Biol.

[16] Jäger S, Bucci C, Tanida I, Ueno T, Kominami E, Saftig P, Eskelinen E-L (2004). Role for Rab7 in maturation of late autophagic vacuoles. J Cell Sci.

[17] Bucci C, Thomsen P, Nicoziani P, McCarthy J, van Deurs B (2000). Rab7: a key to lysosome biogenesis. Mol Biol Cell.

[18] Jacomin AC, Bescond A, Soleilhac E, Gallet B, Schoehn G, Fauvarque MO, Taillebourg E (2015). The deubiquitinating enzyme UBPY is required for lysosomal biogenesis and productive autophagy in drosophila. PLoS One.

[19] Mukai A, Yamamoto-Hino M, Awano W, Watanabe W, Komada M, Goto S (2010). Balanced ubiquitylation and deubiquitylation of Frizzled regulate cellular responsiveness to Wg/Wnt. EMBO J.

[20] Xia R, Jia H, Fan J, Liu Y, Jia J (2012). USP8 promotes smoothened signaling by preventing its ubiquitination and changing its subcellular localization. PLoS Biol.

[21] Balut CM, Loch CM, Devor DC (2011). Role of ubiquitylation and USP8-dependent deubiquitylation in the endocytosis and lysosomal targeting of plasma membrane KCa3.1.. FASEB J.

[22] Berlin I, Higginbotham KM, Dise RS, Sierra MI, Nash PD (2010). The deubiquitinating enzyme USP8 promotes trafficking and degradation of the chemokine receptor 4 at the sorting endosome. J Biol Chem.

[23] Hasdemir B, Murphy JE, Cottrell GS, Bunnett NW (2009). Endosomal deubiquitinating enzymes control ubiquitination and down-regulation of protease-activated receptor 2. J Biol Chem.

[24] Mizuno E, Iura T, Mukai A, Yoshimori T, Kitamura N, Komada M (2005). Regulation of epidermal growth factor receptor down-regulation by UBPY-mediated deubiquitination at endosomes. Mol Biol Cell.

[25] Niendorf S, Oksche A, Kisser A, Löhler J, Prinz M, Schorle H, Feller S, Lewitzky M, Horak I, Knobeloch K-P (2007). Essential role of ubiquitin-specific protease 8 for receptor tyrosine kinase stability and endocytic trafficking in vivo. Mol Cell Biol.

[26] Zhou R, Tomkovicz VR, Butler PL, Ochoa LA, Peterson ZJ, Snyder PM (2013). Ubiquitin-specific peptidase 8 (USP8) regulates endosomal trafficking of the epithelial Na + channel. J Biol Chem.

[27] Pignoni F, Zipursky SL (1997). Induction of Drosophila eye development by decapentaplegic. Development.

[28] Chen MS, Obar RA, Schroeder CC, Austin TW, Poodry CA, Wadsworth SC, Vallee RB (1991). Multiple forms of dynamin are encoded by shibire, a Drosophila gene involved in endocytosis. Nature.

[29] Le Bras S, Rondanino C, Kriegel-Taki G, Dussert A, Le Borgne R (2012). Genetic identification of intracellular trafficking regulators involved in Notch-dependent binary cell fate acquisition following asymmetric cell division. J Cell Sci.

[30] Raiborg C, Stenmark H (2009). The ESCRT machinery in endosomal sorting of ubiquitylated membrane proteins. Nature.

[31] Valentine M, Hogan J, Collier S (2014). The drosophila Chmp1 protein determines wing cell fate through regulation of epidermal growth factor receptor signaling. Dev Dyn.

[32] Press B, Feng Y, Hoflack B, Wandinger-Ness A (1998). Mutant Rab7 causes the accumulation of cathepsin D and cation-independent mannose 6-phosphate receptor in an early endocytic compartment. J Cell Biol.

[33] Vitelli R, Santillo M, Lattero D, Chiariello M, Bifulco M, Bruni CB, Bucci C (1997). Role of the small GTPase Rab7 in the late endocytic pathway. J Biol Chem.

[34] Pulipparacharuvil S, Akbar MA, Ray S, Sevrioukov EA, Haberman AS, Rohrer J, Krämer H (2005). Drosophila Vps16A is required for trafficking to lysosomes and biogenesis of pigment granules. J Cell Sci.

[35] Gutierrez MG, Munafó DB, Berón W, Colombo MI (2004). Rab7 is required for the normal progression of the autophagic pathway in mammalian cells. J Cell Sci.

[36] Huang R, Xu Y, Wan W, Shou X, Qian J, You Z, Liu B, Chang C, Zhou T, Lippincott-Schwartz J (2015). Deacetylation of nuclear LC3 drives autophagy initiation under starvation. Mol Cell.

[37] Nezis IP, Shravage BV, Sagona AP, Lamark T, Bjørkøy G, Johansen T, Rusten TE, Brech A, Baehrecke EH, Stenmark H (2010). Autophagic degradation of dBruce controls DNA fragmentation in nurse cells during late Drosophila melanogaster oogenesis. J Cell Biol.

[38] Nezis IP, Simonsen A, Sagona AP, Finley K, Gaumer S, Contamine D, Rusten TE, Stenmark H, Brech A (2008). Ref(2)P, the Drosophila melanogaster homologue of mammalian p62, is required for the formation of protein aggregates in adult brain. J Cell Biol.

[39] Bartlett BJ, Isakson P, Lewerenz J, Sanchez H, Kotzebue RW, Cumming RC, Harris GL, Nezis IP, Schubert DR, Simonsen A (2011). p62, Ref(2)P and ubiquitinated proteins are conserved markers of neuronal aging, aggregate formation and progressive autophagic defects. Autophagy.

[40] Carre-Mlouka A, Gaumer S, Gay P, Petitjean AM, Coulondre C, Dru P, Bras F, Dezelee S, Contamine D (2007). Control of sigma virus multiplication by the ref(2)P gene of Drosophila melanogaster: an in vivo study of the PB1 domain of Ref(2)P. Genetics.

[41] Mizuno E, Kobayashi K, Yamamoto A, Kitamura N, Komada M (2006). A deubiquitinating enzyme UBPY regulates the level of protein ubiquitination on endosomes. Traffic.

[42] Zhang J, Du J, Lei C, Liu M, Zhu AJ (2014). Ubpy controls the stability of the ESCRT-0 subunit Hrs in development. Development.

[43] Mullins C, Bonifacino JS (2001). The molecular machinery for lysosome biogenesis. Bioessays.

[44] Zeigerer A, Gilleron J, Bogorad RL, Marsico G, Nonaka H, Seifert S, Epstein-Barash H, Kuchimanchi S, Peng CG, Ruda VM (2012). Rab5 is necessary for the biogenesis of the endolysosomal system in vivo. Nature.

[45] Lamb CA, Dooley HC, Tooze SA (2013). Endocytosis and autophagy: shared machinery for degradation. Bioessays.

[46] Razi M, Chan EYW, Tooze SA (2009). Early endosomes and endosomal coatomer are required for autophagy. J Cell Biol.

[47] Ravikumar B, Imarisio S, Sarkar S, O’Kane CJ, Rubinsztein DC (2008). Rab5 modulates aggregation and toxicity of mutant huntingtin through macroautophagy in cell and fly models of Huntington disease. J Cell Sci.

[48] Filimonenko M, Stuffers S, Raiborg C, Yamamoto A, Malerod L, Fisher EM, Isaacs A, Brech A, Stenmark H, Simonsen A (2007). Functional multivesicular bodies are required for autophagic clearance of protein aggregates associated with neurodegenerative disease. J Cell Biol.

[49] Ravikumar B, Moreau K, Jahreiss L, Puri C, Rubinsztein DC (2010). Plasma membrane contributes to the formation of pre-autophagosomal structures. Nat Cell Biol.

[50] Longatti A, Lamb CA, Razi M, Yoshimura S, Barr FA, Tooze SA (2012). TBC1D14 regulates autophagosome formation via Rab11- and ULK1-positive recycling endosomes. J Cell Biol.

[51] Puri C, Renna M, Bento CF, Moreau K, Rubinsztein DC (2013). Diverse autophagosome membrane sources coalesce in recycling endosomes. Cell.

[52] Sarkar S, Carroll B, Buganim Y, Maetzel D, Ng AH, Cassady JP, Cohen MA, Chakraborty S, Wang H, Spooner E (2013). Impaired autophagy in the lipid-storage disorder Niemann-Pick type C1 disease. Cell Rep.

[53] Osellame LD, Rahim AA, Hargreaves IP, Gegg ME, Richard-Londt A, Brandner S, Waddington SN, Schapira AH, Duchen MR (2013). Mitochondria and quality control defects in a mouse model of Gaucher disease--links to Parkinson’s disease. Cell Metab.

[54] Assaker G, Ramel D, Wculek SK, González-Gaitán M, Emery G (2010). Spatial restriction of receptor tyrosine kinase activity through a polarized endocytic cycle controls border cell migration. Proc Natl Acad Sci U S A.

[55] Hennig KM, Colombani J, Neufeld TP (2006). TOR coordinates bulk and targeted endocytosis in the Drosophila melanogaster fat body to regulate cell growth. J Cell Biol.

[56] Scott RC, Schuldiner O, Neufeld TP (2004). Role and regulation of starvation-induced autophagy in the Drosophila fat body. Dev Cell.

[57] Taillebourg E, Gregoire I, Viargues P, Jacomin A-C, Thevenon D, Faure M, Fauvarque M-O (2012). The deubiquitinating enzyme USP36 controls selective autophagy activation by ubiquitinated proteins. Autophagy.

[58] Pircs K, Nagy P, Varga A, Venkei Z, Erdi B, Hegedus K, Juhasz G (2012). Advantages and limitations of different p62-based assays for estimating autophagic activity in Drosophila. PLoS One.

[59] Schindelin J, Arganda-Carreras I, Frise E, Kaynig V, Longair M, Pietzsch T, Preibisch S, Rueden C, Saalfeld S, Schmid B (2012). Fiji: an open-source platform for biological-image analysis. Nat Methods.

[60] Jacomin AC, Nezis IP (2016). Using fluorescent reporters to monitor autophagy in the female germline cells in drosophila melanogaster. Methods Mol Biol.

[61] Bolte S, Cordelieres FP (2006). A guided tour into subcellular colocalization analysis in light microscopy. J Microsc.

