# Supplementary figures and images for: A functional endosomal pathway is necessary for lysosome biogenesis in Drosophila
Source: BMC Cell Biol. 2016 Nov 16;17:36. doi: 10.1186/s12860-016-0115-7 (PMC5112658; doi:10.1186/s12860-016-0115-7)

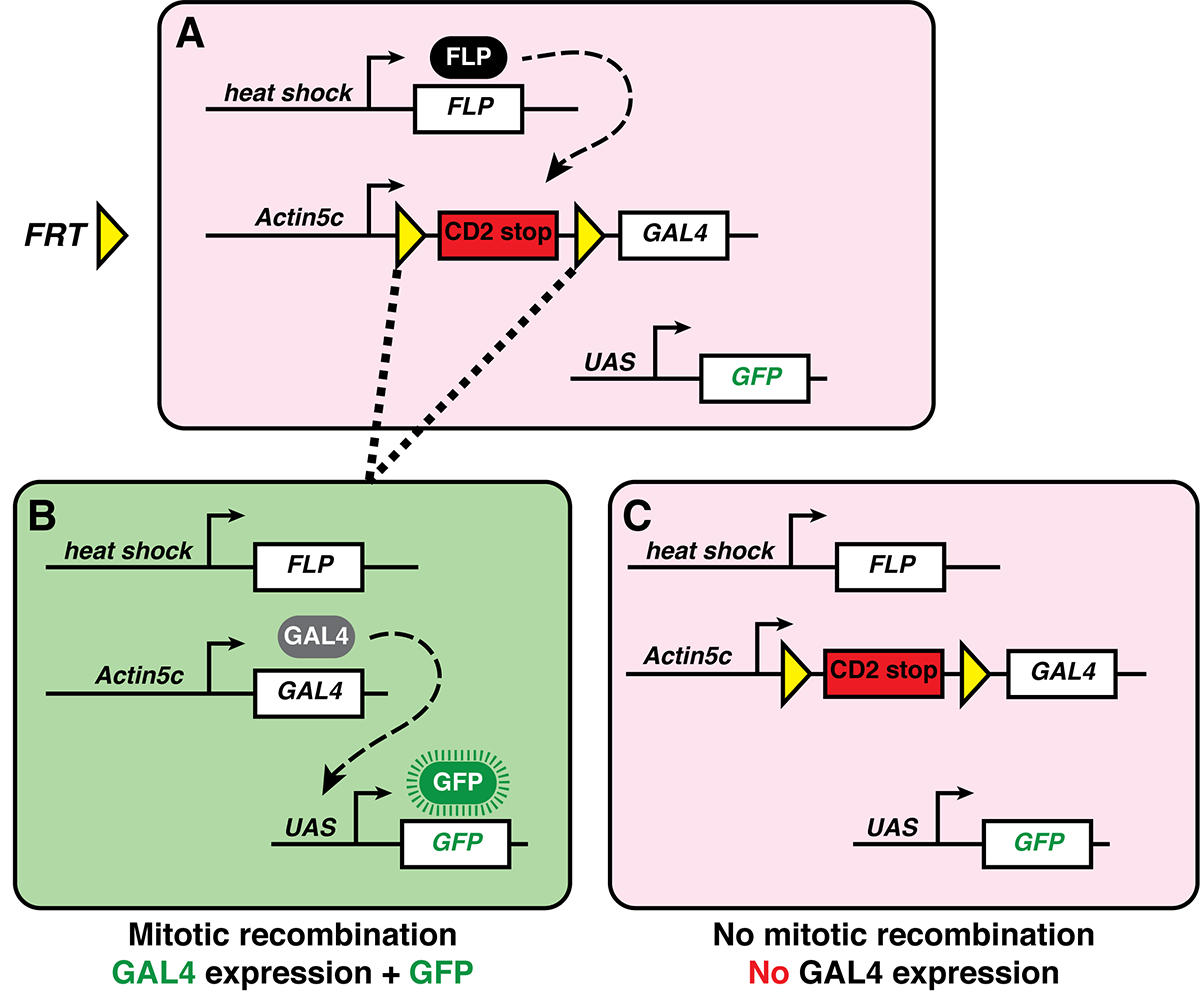

Supplement: Additional file 1: Figure S1. — The FLPout system in Drosophila. (A-B) The recombination between FRT sites by the FLP recombinase under the control of a heat shock promoter (A) results in excision of the CD2- STOP cassette and expression of GAL4 (B) which in turn activates the expression of the transgenes downstream the UAS promoter, including a fluorescent reporter (GFP, or GFP-tagged protein). No recombination between the FRT leaves the CD2-STOP cassette in place, thus preventing GAL4 expression (C). (TIF 676 kb) [file 12860_2016_115_MOESM1_ESM.tif]

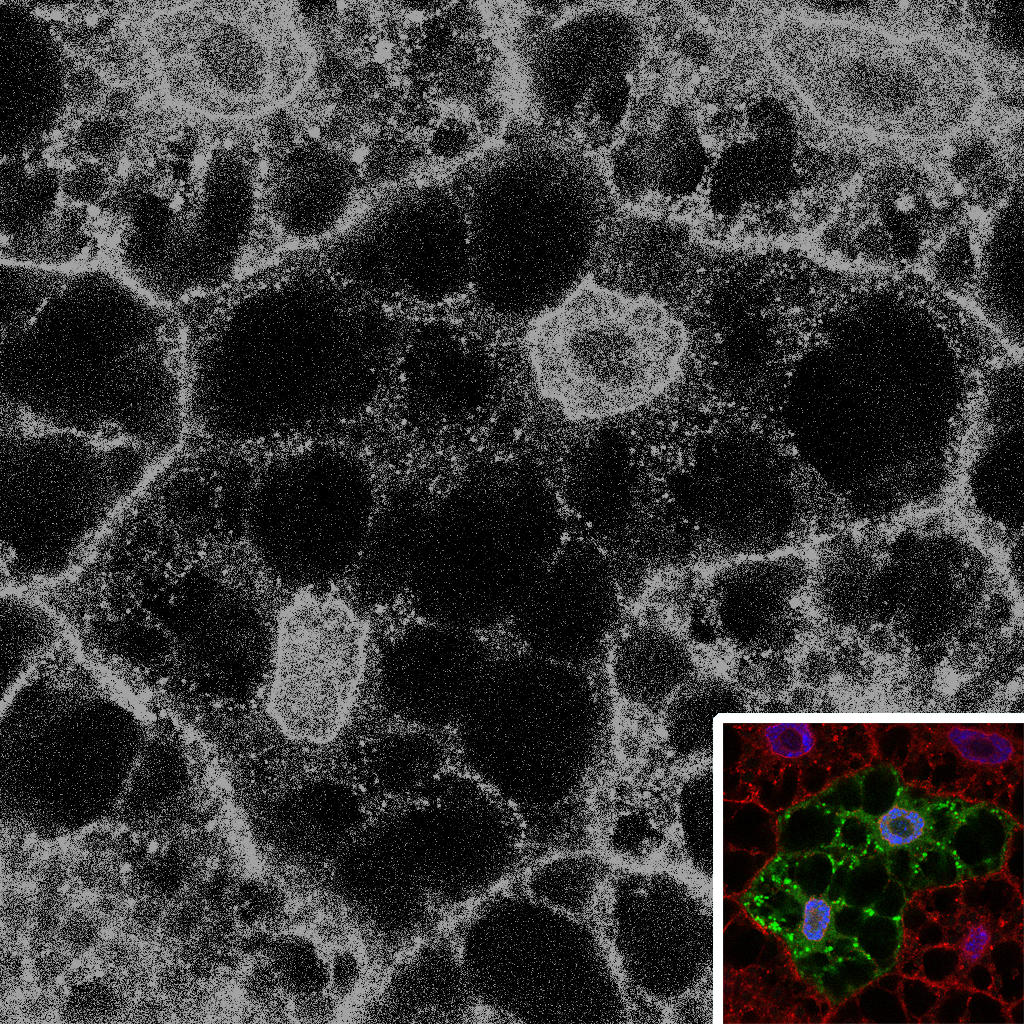

Supplement: Additional file 2: Figure S2. — Validation of the Rab5-IR transgene by immunofluorescence. Confocal sections of larval fat bodies clonally expressing the RNAi against Rab5 stained for Rab5 (red). Fixed fat bodies were additionally stained with Hoechst (blue). The silenced cells were identified by the expression of the GFP-Atg8a transgene (green). Genotype: y w hs-FLP/+; UAS-GFP- Atg8a/+; Ac > CD2 > Gal4/UAS-Rab5-IR. (TIF 1509 kb) [file 12860_2016_115_MOESM2_ESM.tif]

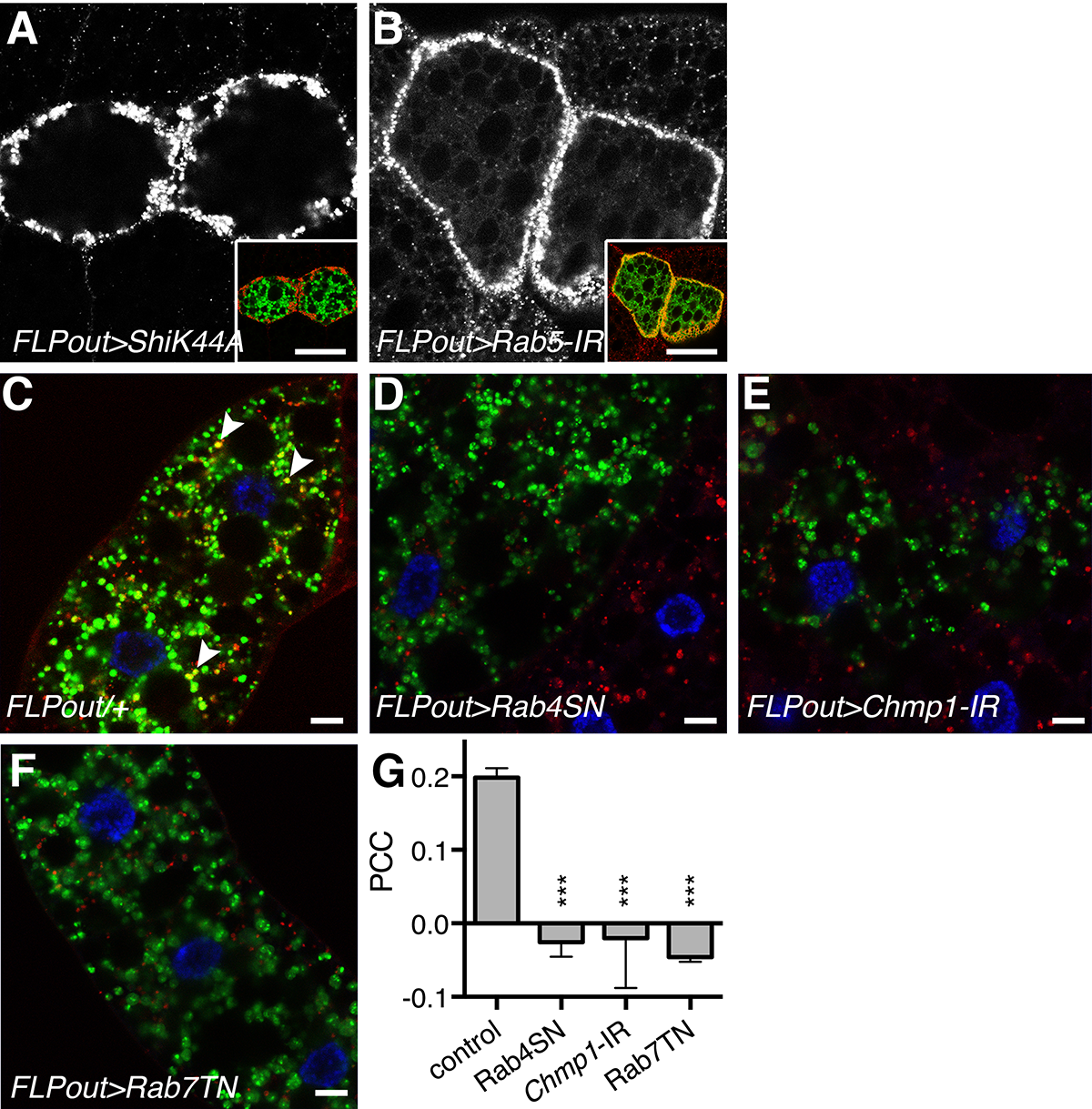

Supplement: Additional file 3: Figure S3. — Validation of defects in the endosomal pathway by Texas Red-Avidin uptake. (A-B) The endocytic tracer TR-avidin fails to be internalized in clonal cells expressing either ShiK44A (A) or Rab5-IR (B). Clones were detected by the co-expression of the autophagy marker GFP-Atg8a. (C-G) Internalized TR-avidin fails to be transported to the lysosomes when late stages of the endocytic process are defective. Clonal cells were detected by the expression of the lysosomal marker GFP-LAMP1. Occasional colocalization between the endocytic tracer TR-avidin and the lysosomes are observed in control cells (D) but not in cells expressing Rab4SN (E), Chmp1-IR (F) or Rab7TN (G). Quantification of the colocalization between the TR-avidin and GFP-LAMP1 using the Pearson’s Correlation Coefficient (PCC) is shown in C. Bars denote mean ± s.d. Statistical significance was determined using one-way ANOVA: *p < 0.05, **p < 0.005, ***p < 0.0005, ****p < 0.0001. Genotypes: (A) y w hs-FLP/UAS-ShiK44A; UAS-GFP-Atg8a/+; Ac > CD2 > Gal4/ UAS-ShiK44A, (B) y w hs-FLP/+; UAS-GFP-Atg8a/+; Ac > CD2 > Gal4/UAS-Rab5-IR, (C) y w hs-FLP/+; UAS-GFP-LAMP1/+; Ac > CD2 > Gal4/+, (D) y w hs-FLP/+; UAS-GFP-LAMP1/+; Ac > CD2 > Gal4/UAS-Rab4SN, (E) y w hs-FLP/+; UAS-GFP-LAMP1/+; Ac > CD2 > Gal4/UAS-Chmp1-IR, (F) y w hs-FLP/+; UAS-GFP-LAMP1/UAS-Rab7TN; Ac > CD2 > Gal4/+. (TIF 1831 kb) [file 12860_2016_115_MOESM3_ESM.tif]

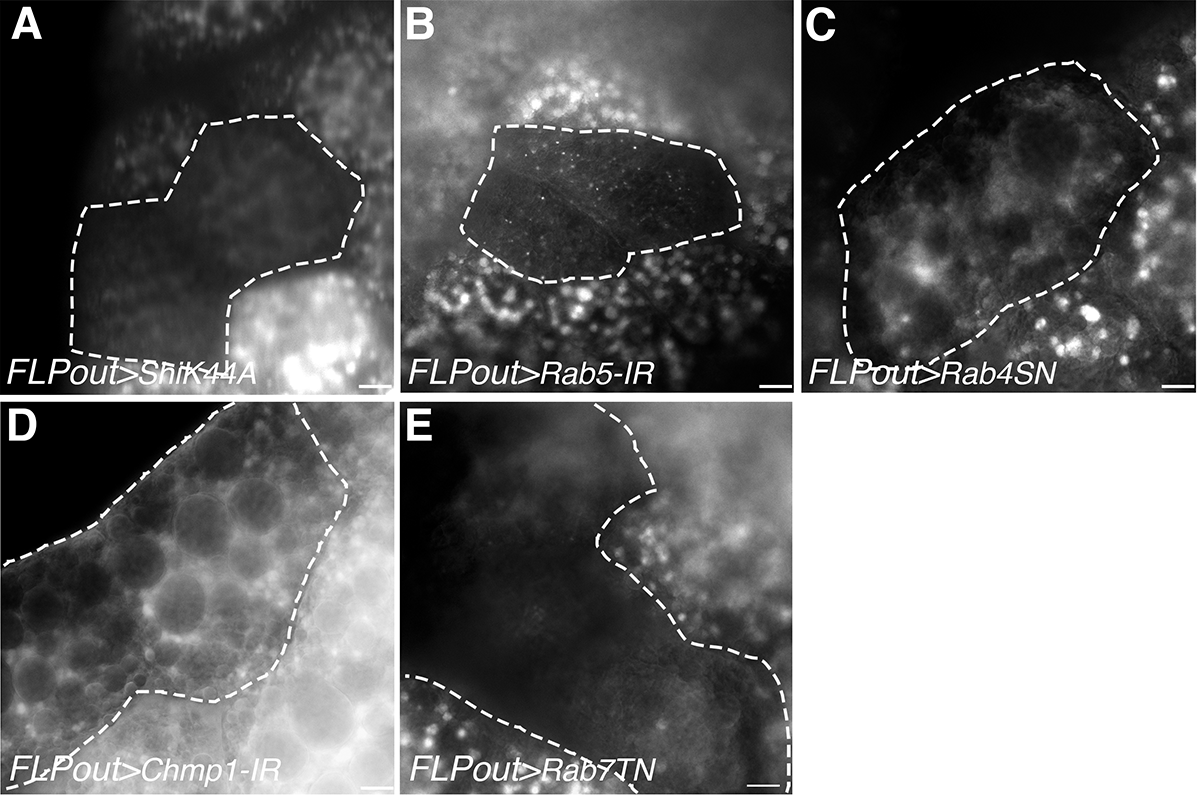

Supplement: Additional file 4: Figure S4. — The endosomal pathway is required for the starvation-induced acidification of the lysosomes. Larvae clonally expressing the dominant negative or silencing transgenes for Shibire (A), Rab5 (B), Rab4 (C), Chmp1 (D) or Rab7 (E) were starved to induce autophagy and the acidification of the lysosomes. Alive fat bodies were stained with Lysotracker-Red. Clonal cells were identified by the expression of the GFP-Atg8a transgene. Genotypes: (A) y w hs-FLP/UAS-ShiK44A; UAS-GFP-Atg8a/+; Ac > CD2 > Gal4/ UAS-ShiK44A, (B) y w hs-FLP/+; UAS-GFP- Atg8a/+; Ac > CD2 > Gal4/UAS-Rab5-IR, (C) y w hs-FLP/+; UAS-GFP- Atg8a/+; Ac > CD2 > Gal4/UAS-Rab4SN, (D) y w hs-FLP/+; UAS-GFP- Atg8a/+; Ac > CD2 > Gal4/UAS-Chmp1-IR, (E) y w hs-FLP/+; UAS-GFP- Atg8a/UAS-Rab7TN; Ac > CD2 > Gal4/+. (TIF 791 kb) [file 12860_2016_115_MOESM4_ESM.tif]
